# Phototoxic effects of two common marine fuels on the settlement success of the coral *Acropora tenuis*

**DOI:** 10.1038/s41598-018-26972-7

**Published:** 2018-06-05

**Authors:** F. Mikaela Nordborg, Florita Flores, Diane L. Brinkman, Susana Agustí, Andrew P. Negri

**Affiliations:** 1 Australian Institute of Marine Science, Townsville, 4810 Queensland Australia; 2 Department of Marine Sciences, University of Gothenburg, Göteborg, 40530 Sweden; 3 Red Sea Research Centre, King Abdullah University of Science and Technology, Biological Environmental Science and Engineering Division, Thuwal, 23955 Saudi Arabia; 4 AIMS@JCU, Division of Research & Innovation, James Cook University and Australian Institute of Marine Science, Townsville, 4810 Queensland Australia

## Abstract

Coral reefs are at risk of exposure to petroleum hydrocarbons from shipping spills and uncontrolled discharges during extraction. The toxicity of petroleum hydrocarbons can substantially increase in the presence of ultraviolet radiation (UVR), therefore spills in shallow coral reef environments may be particularly hazardous to reef species. Here we investigated the sensitivity of coral larvae (*Acropora tenuis*) to dissolved hydrocarbons from heavy fuel oil (HFO) and diesel in the absence and presence of UVR. Larval settlement success decreased with increasing concentrations of dissolved HFO, and co-exposure to UVR doubled the toxicity: 50% effect concentrations (EC_50_) decreased from 96 (−UVR) to 51 (+UVR) total petroleum aromatic hydrocarbons (TPAH). Toxic thresholds for HFO were similar to concentrations reported during marine spills: EC_10_s of 24 (−UVR) and 15 (+UVR) µg l^−1^. While less toxic, diesel also reduced settlement and exhibited phototoxicity: EC_10_s of 122 (+UVR) and 302 (−UVR) µg l^−1^. This study demonstrates that the presence of UVR increases the hazard posed by oil pollution to tropical, shallow-water coral reefs. Further research on the effects of oils in the presence of UVR is needed to improve the environmental relevance of risk assessments and ensure appropriate protection for shallow reef environments against oil pollution.

## Introduction

### Petroleum hydrocarbons in marine environments

Petroleum hydrocarbons are considered among the most prominent pollution threats to marine environments^1^; however, the risks they pose to coral reef ecosystems remain poorly understood^2,3^. The environmental effects resulting from oil spills and uncontrolled discharges from extraction vary widely and are dependent on a large number of factors^4^. Hydrocarbon concentrations in marine environments have been measured following large scale spills^5–7^, with dissolved hydrocarbon concentrations ranging between 22 and 189 µg l^−1^ total polycyclic aromatic hydrocarbons (PAH)^5,8,9^ and up to 10,600 µg l^−1^ total recoverable hydrocarbons (TRH)^10^. Hydrocarbons can be retained within an ecosystem during spill events and, despite natural dilution and degradation, can remain detectable long after the spill has ended^1^.

The toxicity of petroleum hydrocarbons to most marine species is predominantly related to the water soluble components, which largely consist of the monoaromatic hydrocarbons (MAH) and PAHs^11^. PAHs, in particular, are considered acutely toxic to aquatic biota^12,13^, with toxic threshold concentrations for PAHs often orders of magnitude lower than those of MAHs^14^. However, the overall toxicity of dissolved petroleum hydrocarbon from spills is also dependent on the relative concentrations of each component^15^. Aromatic hydrocarbons in petroleum oils are classified as type I narcotic chemicals^14^. Assuming the same mode of toxic action, the total toxicity of the water soluble components of an oil can be predicted using the narcotic target lipid model (NTLM), which sums the expected toxicity and concentration of each aromatic component^14^ (see Methods).

### Phototoxicity of PAHs

The toxicity of the dissolved aromatic mixtures that result from oil spills may increase in the presence of UVR due to the phototoxic contribution of some PAHs^12^. PAH phototoxicity occurs through the formation of radical oxygen species and/or transformation of PAHs into more toxic photoproducts^12,13^. Co-exposure of PAHs and UVR can increase the toxicity of individual PAHs 1000-fold^16^; however, the potential for phototoxicity depends on the compound’s stability, radiation absorbance properties^12^, the type of UVR exposure^12,17^ and the exposed organism’s sensitivity^17^. The potential for harmful effects to marine organisms is higher for UVA (320–400 nm) than UVB (280–320 nm) as the absorption maxima (hence, photoactivation) of many PAHs fall within the UVA range^12,18^, and UVB is more strongly attenuated in seawater^19^. The penetration of UVR in marine environments is also dependent on a range of physical and biological factors^19^, and tropical oligotrophic coral reefs may be at a particularly high risk of PAH phototoxicity as reef organisms are frequently exposed to high solar radiation, including UVR^13,18^. PAH phototoxicity is not always taken into consideration for risk assessments and management unless the ecological relevance, including UVR exposure and spectral profiles, in the ecosystem have been characterised^13,20,21^. However, it is increasingly recognised that a considerable proportion of UVR penetrates to ecologically relevant depths in some marine environments, including coral reefs^18,22–24^ and that exposure to cumulative pressures, such as pollution and UVR, can result in increased environmental impacts^13,18,25^.

### Petroleum hydrocarbon and PAH toxicity to corals

Despite a renewed demand for marine hydrocarbon toxicity research following the Deepwater Horizon spill^26^, significant knowledge gaps on the potential effects of hydrocarbon exposure to corals remain^18,27–29^ and the sensitivity of tropical marine species to hydrocarbons is relatively understudied^14,30^. Investigations into the effects of petroleum hydrocarbons, including PAHs, on coral indicate that negative impacts can occur at concentrations as low as 2–20 µg l^−1^ total hydrocarbons (THC)^31^. However, inconsistencies in exposure methodologies, toxic endpoints and reported toxicity values make comparisons between studies problematic^2,15,18,32^. This issue is further compounded by the failure of many studies to present the chemical composition of treatment solutions, in particular the more soluble and toxic MAHs and PAHs^15^. However, the studies that have been conducted show that hydrocarbons can be toxic to all life history stages of coral and that larval settlement is generally more sensitive than fertilization^33^, larval survival^7,33,34^ or the health and survival of adult corals^35^. The sensitive larval settlement process is recognised as an ecologically relevant endpoint due to its importance in the recruitment process and subsequent maintenance of adult populations^29,36^. The larval life stages of aquatic animals may also be at higher risk than adults to phototoxic effects due to their small size, often transparent bodies, and time spent in shallow waters^16^.

Four studies have investigated phototoxic effects of petroleum hydrocarbons on corals and each indicated that their sensitivity to dissolved aromatics may increase with co-exposure to UVR (summarised in Table 1). However, the majority of laboratory studies exposing corals to hydrocarbons have not included co-exposure to UVR^2^, so the impacts of hydrocarbon pollution on coral reefs may be significantly underestimated in the context of likely high UVR exposure *in situ*. To assess the potential for UVR to increase the sensitivity of coral larvae to spills of heavy fuel oil (HFO) and diesel we: (i) assessed UVR irradiance on one inshore and one mid-shelf reef on the Great Barrier Reef (GBR; Australia); (ii) characterised the chemical composition of the two fuels and their water accommodated fractions (WAFs); and (iii) predicted their narcotic toxicity to marine species using the NTLM. We then (iv) exposed larvae of the reef building coral *Acropora tenuis* (Dana, 1846) to HFO and diesel WAFs in the absence and presence of UVR (±UVR), at intensities similar to those encountered on the GBR, and assessed the ability of exposed larvae to successfully complete settlement and metamorphosis into sessile polyps following each treatment.

**Table 1:** Summary of previous studies of the phototoxic effects of petroleum hydrocarbons on scleractinian corals.

Species	Hydrocarbon	UVR source	Chemical analysis	Endpoint	Toxicity values (µg l^−1^)	Phototoxic effects	Reference
*Acropora tenuis* (larvae)*	Anthracene (A), Phenanthrene (P) (48 h exposure)	Artificial UVR 0.68 mW cm^−2^ (10 h per 24 h, co-exposure)	PAH (GC-MS)	Survival	*LC*_50_: 44 (−UVR) and 18 (+UVR) µg l^−1^ (A); no effect (±UVR) (P)	Yes: Anthracene	Overmans *et al*.^18^
				Metamorphosis	*EC*_50_: 45 (−UVR) and 6.3 (+UVR) µg l^−1^ (A); 91 (−UVR) and 66 (+UVR) µg l^−1^ (P)	No: Phenathrene	
*Acropora tenuis* (larvae)	Australian North West Shelf condensate (24 h exposure)	Ambient solar 4.5–6.8 mW cm^−2^ (2 h, co-exposure)	BTEX, PAH, TRH (GC-MS)	Metamorphosis	*IC*_50_: 339 (−UVR) and 132 (+UVR) µg TPAH l^−1^	Yes	Negri *et al*.^29^
*Porites divaricata* (adult)	Fluoranthene (4.5 h exposure)	Ambient solar (ambient L:D cycle for 6 d, seq. exposure)	Nil	Mortality/Bleaching	*LC*_50_: 435.2 (−UVR; lower side of branches) and 31.4 µg l^−1^ (+UVR; upper side of branches)	Yes	Carmen Guzmán Martínez *et al*.^27^
*Fungia scutaria* (larvae)	Pyrene (2 h exposure)	Ambient solar 0.41–1.4 mW cm^−2^ (up to 8 h, seq. exposure)	Nil	Mortality	*F*. *scutaria* (+UVR): 100% mortality ≤1 h after exposure to 48 µg l^−1^.	Yes: *F*. *scutaria* *M*. *verrucosa* *P*. *damicornis*	Peachey & Crosby^17^
*Montipora verrucosa* (adult)					*M*. *verrucosa* (+UVR): bleached 24 h after exposure to ≥16 µg l^−1^.		
*Pocillopora damicornis* (adult)				Bleaching	*P*. *damicornis* (+UVR): bleached 24 h after exposure to 48 µg l^−1^.	No: *P*. *compressa*	
*Porites compressa* (adult)					*P*. *compressa* (+UVR): no effect.		
					All species (−UVR): no effect.		
*Montipora verrucosa* (adult)	Pyrene (2 h exposure)	Artificial UVR 0.98–1.0 mW cm^−2^ (up to 8 h, seq. exposure)	Nil	Bleaching	Not reported	No (both species)	
*Pocillopora damicornis* (adult)							

Study methodology, species tested, chemical analysis performed (if applicable), toxic endpoint and main results shown for each study. If no threshold values or concentrations are presented no effect was observed. Seq = sequential, organisms first exposed to pollutant followed by exposure to UVR while kept in clean FSW. GC-MS = gas chromatography-mass spectrometry. Nil = no analysis reported.

*Stress response-related gene expression and enzyme activity were also investigated (see reference for further details).

## Results

### Chemical analysis

Neat HFO consisted primarily of higher molecular weight hydrocarbons while neat diesel contained a higher proportion of BTEX (benzene, toluene, ethylbenzene and xylene) and other lower molecular weight hydrocarbons (Figs S-1 and S-2, Supplementary information). ∑PAH in HFO (constituting 99.6% of TPAH) was almost 10-fold higher than in diesel (79% of TPAH), whereas ∑BTEX was ~8-fold lower in HFO compared to diesel (see summary in Table 2 and detailed results in Table S-1, Supplementary information). Phenols were below the limit of quantitation in both oils (Table S-1, Supplementary information). Freshly prepared 100% HFO and diesel WAFs contained similar TPAH concentrations (930 and 913 µg l^−1^, respectively), but the proportions of ∑BTEX and ∑PAH varied (Table S-2, Supplementary information). While HFO WAF contained almost equal concentrations of ∑BTEX and ∑PAH (52% and 48% of TPAH, respectively), ∑BTEX dominated the diesel WAF (98% of TPAH). The most abundant PAHs in the WAFs were naphthalene, alkylnaphthalenes, fluorene and phenanthrene; the HFO WAF also contained acenaphthene and dibenzothiophene (Table S-2, Supplementary information). The concentration of TPAH in fuel WAFs decreased by up to 34% over the 48 h exposure (Tables 2 and S-2, Supplementary information). Comparisons of the observed and predicted concentrations of 1-, 2- and 3-ring compounds in the freshly prepared, undiluted WAFs (Tables S-2 and S-3, Supplementary information) as per Redman *et al*.^15^ indicated that no oil droplets were present in either HFO or diesel WAFs.

**Table 2:** Time-averaged concentrations of ∑BTEX, ∑PAH and TPAH in undiluted fuel WAFs, ∑BTEX, ∑PAH and TRH in neat fuels, and toxic units (narcosis) calculated from predicted and observed fuel WAF concentrations.

		[WAF] (µg l^−1^)			[Neat fuel] (mg kg^−1^)			*TU*_*WAF*_			*TU*_*Neat fuel*_		
		*∑BTEX*	*∑PAH*	*TPAH*	*∑BTEX*	*∑PAH*	*TPAH*	*∑BTEX*	*∑PAH*	*TPAH*	*∑BTEX*	*∑PAH*	*TPAH*
HFO	−*UVR*	498 (+4%)	440 (−1%)	938 (+2%)	190	50494	50684	0.03	0.43	0.46	0.01	0.91	0.92
	+*UVR*	478 (−4%)	414 (−13%)	892 (−8%)				0.03	0.40	0.43			
Diesel	−*UVR*	745 (−33%)	14 (−57%)	759 (−34%)	1491	5723	7214	0.04	0.01	0.05	0.07	0.12	0.19
	+*UVR*	767 (−28%)	16 (−41%)	783 (−29%)				0.05	0.02	0.06			

Time-averaged concentrations per light treatment calculated from concentrations measured at t_0h_ and t_48h_ for HFO and diesel WAFs; % change in concentrations after 48 h indicated in brackets. TUs calculated using an average CTLBB value (86.8 µmol g^−1^ octanol; n = 15) and aqueous concentrations of BTEX and PAH observed in fuel WAFs (TU_WAF_) or predicted from neat fuel oil concentrations (TU_Neat fuel_). TPAH = total petroleum aromatic hydrocarbons, ∑PAH = sum of individual PAH concentrations, ∑BTEX = sum of benzene, toluene, ethylbenzene and xylene concentrations. For full analytical results see Tables S-1 and S-2, Supplementary information.

### Ultraviolet radiation intensities

**Figure 1:**
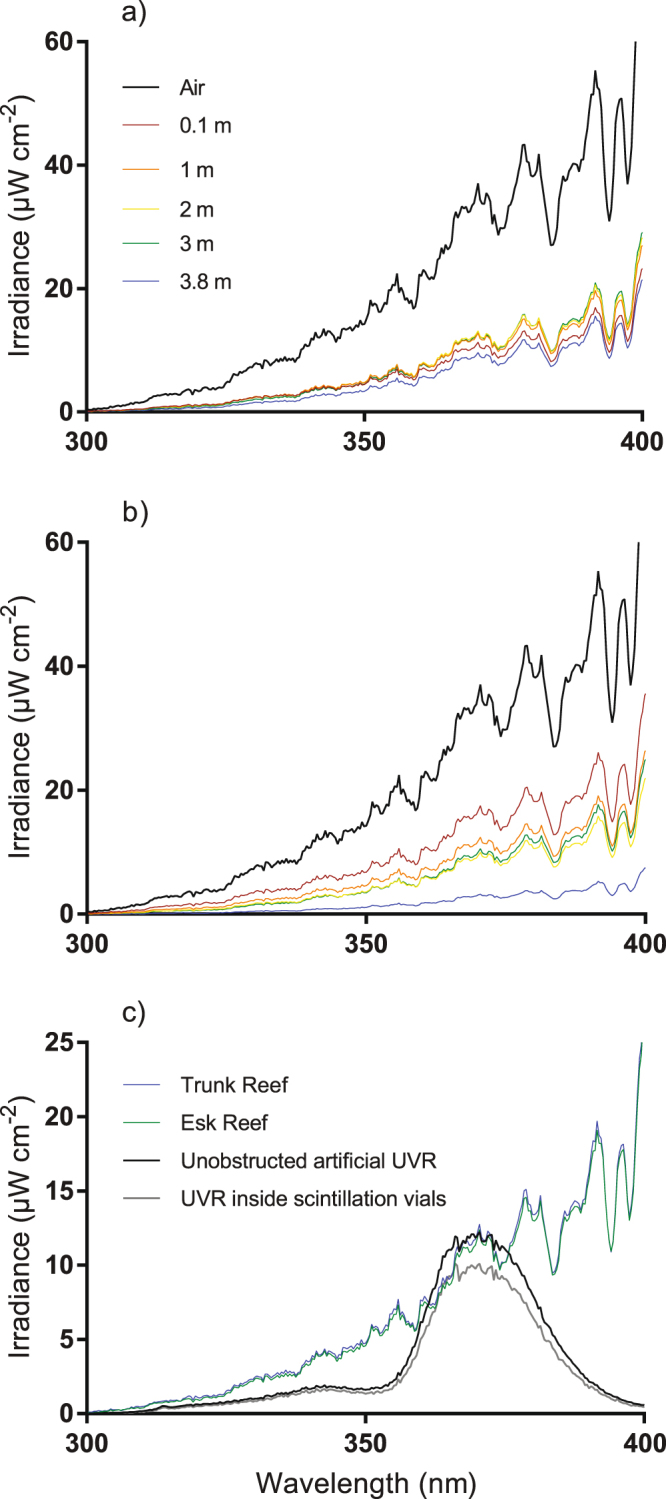
Penetration of ultraviolet radiation (UVR) on Trunk Reef (**a**) and Esk Reef (**b**) as well as a comparison (**c**) of exposure intensity and spectrum of artificial UVR used during settlement toxicity assays and UVR observed *in situ* on the Great Barrier Reef (GBR; Australia) during spring. Full spectrum measurements of UVR in air and at 0.1, 1, 2, 3, and 3.8 m depth at a mid-shelf (Trunk reef) and inshore (Esk reef) reef on the GBR in October 2016. Total irradiance values calculated using the percentage reductions in light intensity recorded for each wavelength at each depth (in relation to measurements in air) and total irradiance measurements in air on a clear day (cloud coverage <5%). Comparison of UVR intensity and spectrum emitted from fluorescent tubes used in settlement toxicity assays, calculated UVR exposure inside scintillation vials and UVR exposures observed at 1 m depth on the GBR during spring.

The spectral profiles for both the Trunk Reef (mid-shelf) and Esk Reef (inshore) sites showed that the largest decrease in irradiance occurred when light passed from air to water (Fig. 1a and b). Attenuation of UVR at Esk Reef was somewhat lower than at Trunk Reef for shallow measurement depths, despite the higher turbidity (0.1 and 0.8 nephelometric turbidity units, NTU, respectively). However, irradiance was attenuated less with depth at Trunk Reef and the irradiance for the deepest measurements was much higher at Trunk Reef than at Esk Reef (Fig. 1a and b). The depth which 10% of UVR irradiance penetrated to (Z10%) for 305 and 340 nm at Trunk Reef was ~6 and 7 m, respectively. On Esk Reef, Z10% was ~2.7 and 2.4 m for 305 and 340 nm, respectively.

Experimental lights, when positioned 170 mm from the sensor, emitted UVA radiation similar to the irradiance observed at 1 m depth on Trunk Reef and Esk Reef (Fig. 1c). Average total UVA and UVB (280–400 nm) radiation was 0.9 mW cm^−2^ (SE = 0.16). Attenuation by the glass scintillation vials used in the experiment reduced the average absolute irradiance by ~17% for wavelengths between 300–400 nm (Fig. 1c; measured using Jaz spectrometer calibrated according to manufacturer’s recommendations) giving a calculated total irradiance between 280–400 nm of approximately 0.75 mW cm^−2^ inside the scintillation vials.

### Larval settlement assays

**Figure 2:**
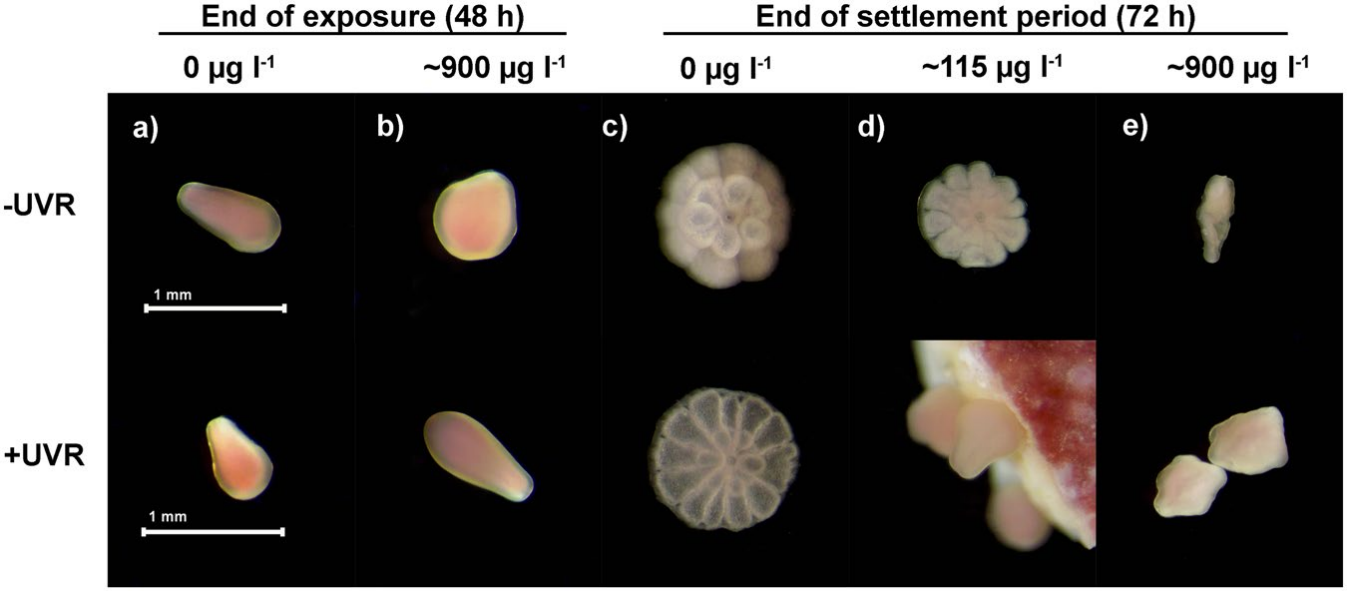
Photomicrographs of *A*. *tenuis* planulae larvae exposed to water accommodated fractions (WAFs) of heavy fuel oil (HFO) in the absence (−UVR) or presence (+UVR) of ultraviolet radiation as well as juvenile polyps (following settlement). Larvae exposed to (**a**) filtered seawater (0 µg TPAH l^−1^), (**b**) approximately 900 µg TPAH l^−1^ after 48 h of exposure as well as juvenile polyps, attached and unattached larvae treated with (**c**) FSW (0 µg TPAH l^−1^), (**d**) 115 µg TPAH l^−1^ and (**e**) approximately 900 µg TPAH l^−1^ HFO WAF after 48 h of exposure, introduction of settlement inducer and a 24 h settlement period (a total ~72 h after experiment start). All concentrations in µg TPAH l-1. TPAH = total petroleum aromatic hydrocarbons.

Temperature was maintained at 27.6 ± 1.4 °C (mean ± SD) in the experiments while photosynthetically active radiation (PAR) in the +UVR and −UVR treatments averaged 0.95 ± 0.10 and 1.23 ± 0.10 mW cm^−2^, respectively. Dissolved oxygen concentration averaged 7.9 ± 0.33 mg l^−1^ with all replicates maintaining concentrations >7.0 mg l^−1^ for the duration of the exposure period while pH and salinity averaged 8.1 ± 0.06 and 37.0 ± 0.32 psu, respectively. *A*. *tenuis* larvae in control treatments were observed to actively swim throughout the exposure (Fig. 2a), but swimming behaviour was not assessed in the fuel exposure treatments. In the control treatments, an average of 73% (SE = 4) larvae underwent settlement in the presence of CCA chips within 24 h (Fig. 2c). This level of settlement success did not change in the presence of UVR with 77% (SE = 3.5) of larvae successfully undergoing settlement. Average larval settlement ≥70% in control treatments was considered indicative of a normal response to settlement inducers based upon several previous studies using CCA or extracts of CCA to initiate settlement of *Acropora* spp^29,37,38^.

### Heavy fuel oil toxicity

**Figure 3:**
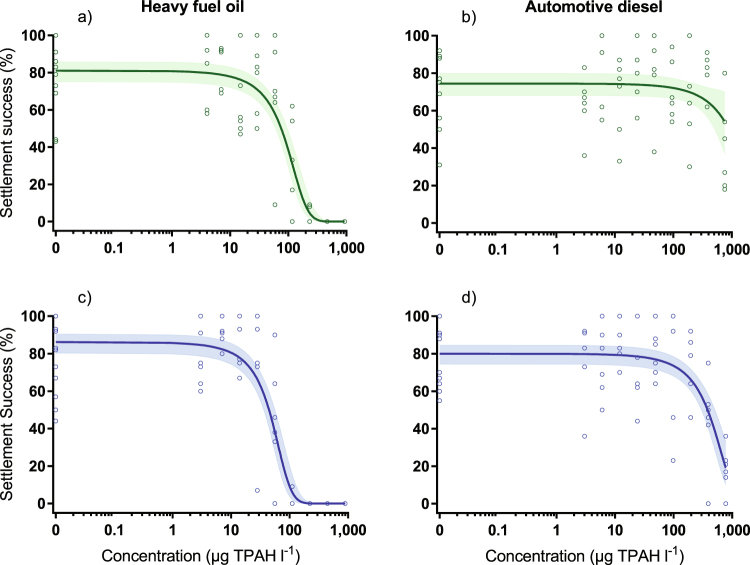
Concentration-response curves for coral larval settlement following exposure to heavy fuel oil (**a** and **c**) and diesel (**b** and **d**) water accommodated fractions (WAF) in the presence (blue) and absence (green) of ultraviolet radiation (µg TPAH l^−1^). Model mean (solid line) and 95% confidence intervals (shaded area) for quasibinomial GLMs fitted for the settlement success data of each treatment combination as well as observed settlement success for each replicate (open ring) used in model fitting. All concentrations in µg l^−1^. n_HFO+/−UVR_ = 63, n_diesel−UVR_ = 65, n_diesel+UVR_ = 64. TPAH = total petroleum aromatic hydrocarbons.

HFO WAF inhibited larval settlement in both the absence and presence of UVR (Fig. 3a,c and Table 3). Little or no effect on settlement success was observed at low concentrations (<10 µg l^−1^ TPAH); above which, settlement decreased with increasing TPAH concentration (Fig. 3). The toxicity of HFO WAF was enhanced in the presence of UVR resulting in a ~50% decrease of the EC_50_ from 96 to 51 µg l^−1^ TPAH (95% confidence intervals did not overlap; Table 3). The toxic threshold value (EC_10_) also reduced in the presence of UVR (Table 3). Obvious mortality (disintegrating cell membranes) was only observed in the highest TPAH treatment (890 µg l^−1^), in the presence of UVR, at the end of the 48 h exposure. No other visible effects on larvae, in either control or HFO WAF treatments, were observed and surviving larvae in the highest HFO treatments exhibited normal morphology (Fig. 2a and b). At 72 h (following the 24 h settlement period) unattached larvae and attached juvenile polyps in control treatments retained normal morphology (Fig. 2c). At this point the frequency and severity of abnormalities increased with increasing TPAH concentration in attached juvenile polyps (asymmetrical or underdeveloped recruits; Fig. 2d) and non-settled larvae (bumps, deformities and necrosis; Fig. 2e) for both ±UVR treatments. Severe deformities were observed in unattached larvae at TPAH concentrations as low as 28 µg l^−1^ (+UVR) and a substantial proportion of larvae were immobile and/or dead in the highest concentration treatment (890 µg l^−1^). Most of the successfully attached juvenile polyps underwent complete metamorphosis in the absence of UVR; however, larvae exposed to HFO WAFs appeared to develop more slowly than expected and some had only undergone partial metamorphosis in higher concentration treatments at the time of assessment.

**Table 3:** Concentrations of TPAH in fuel WAFs which inhibited 50% (EC_50_) and 10% (EC_10_) of larval settlement in the absence (−UVR) and presence (+UVR) of ultraviolet radiation.

		*EC*_10_	*EC*_50_	*Approximate r* ^2^	*Deviance*	*df*
HFO	−UVR	24 (9–39)	96 (75–116)	0.748	146.12	61
	+UVR	15 (6–23)	51 (41–62)	0.767	137.03	61
Diesel	−UVR	302 (51–552)	~1300* (253–2410)	0.062	196.07	63
	+UVR	122 (50–194)	494 (371–617)	0.423	165.97	62

Effect concentrations calculated from settlement success data fitted with quasibinomial GLMs with approximate r^2^, deviance and degrees of freedom (df) shown for each treatment combination. 95% confidence intervals in brackets where applicable. All concentration values in µg TPAH l^−1^. TPAH = total petroleum aromatic hydrocarbons. *Extrapolated from fitted model.

### Diesel toxicity

Diesel WAF also inhibited larval settlement, but little effect was observed at low to moderate concentrations (<100 µg l^−1^ TPAH) regardless of UVR treatment (Fig. 3). In the absence of UVR, only the highest treatment concentration (759 µg l^−1^ TPAH) inhibited settlement success. An approximate EC_50_ of ~1300 µg l^−1^ TPAH (without confidence intervals), was estimated by extrapolation (Fig. 3b and Table 3). UVR greatly enhanced the toxiciy of diesel WAF and decreased the EC_50_ to 494 µg l^−1^ TPAH (Fig. 3d and Table 3). At moderate to high TPAH concentrations (≥200 µg l^−1^) underdeveloped juvenile polyps were observed in the presence of UVR, and the few attached juvenile polyps observed in the highest concentration (782 µg l^−1^ TPAH) were either underdeveloped or abnormal. In the absence of UVR, some underdeveloped and malformed attached juvenile polyps were also observed; however, fully metamorphosed polyps were still present at the highest concentration tested (758 µg l^−1^ TPAH). Some minor abnormalities (e.g. bends and lumps) were observed in unattached larvae from ± UVR treatments but with no apparent relationship to TPAH concentration.

### Modelled narcotic toxicity

The narcotic toxicities of the undiluted diesel and HFO WAFs used in the present study were estimated using the NTLM^14^. This modelling yielded toxic units (TU) based on both the predicted^39^ and measured concentrations of aromatics in undiluted WAFs. TUs can be used to compare the toxicity of complex mixtures; the greater the TU, the more toxic the solution (see Methods). The narcotic toxicity of HFO and diesel WAFs, calculated using WAF concentrations predicted from neat fuel compositions (TU_Neat fuel_; Table 2)^14^, was primarily attributable to PAHs (TU_∑PAH_ > TU_∑BTEX_; Table 2). For undiluted HFO WAF, 0.92 TU_Neat fuel_ indicates that close to 50% mortality would be expected, while TUs, calculated by applying measured WAF concentrations to the NTLM (TU_WAF_: 0.46 −UVR and 0.43 +UVR), were approximately half that value (Table 2). The total narcotic toxicity for undiluted diesel WAF indicated that relatively low mortality would be expected following exposure, when calculated using predicted WAF concentrations (TU_Neat fuel_: 0.19), or close to zero mortality, when measured WAF concentrations were applied (TU_WAF_: 0.05 −UVR to 0.06 +UVR).

## Discussion

Co-exposure to UVR considerably enhanced the negative impacts of the water-soluble fractions of two petroleum fuels on the settlement success of coral larvae. This phototoxicity was evident under UVR intensities equal to or lower than expected exposures in shallow reef habitats, and the toxic thresholds (EC_10_) for dissolved aromatics occurred at concentrations below those detected after major accidental spills or releases^7–9^. These results indicate that by ignoring phototoxicity, the hazards posed by oil spills to coral larvae may be substantially underestimated in shallow-water tropical reef systems.

Coral larval settlement was very sensitive to HFO WAF with threshold concentrations (EC_10_ and EC_50_) lower than or similar to previously reported concentrations during and after oil spills (42–189 µg l^−1^ ∑PAH^5,8,9^ and 22 µg l^−1^ TPAH^9^). The EC_10_ and EC_50_ values (24 and 96 µg l^−1^ TPAH, respectively; −UVR) were also lower than those reported for inhibition of larval settlement in the same species in 24 h exposures to natural gas condensate in the absence of UVR (103 and 339 µg l^−1^ TPAH, respectively)^29^. Differences in sensitivity are likely to be due to the longer duration of the current exposure and the higher proportion of more toxic PAHs in the HFO WAF. Conversely, the sensitivity of *A*. *tenuis* larvae to diesel WAF in the absence of UVR was less than that reported for either HFO or condensate, potentially due to the lower concentrations of total dissolved PAHs (14 µg l^−1^) in the undiluted diesel WAF compared to HFO (440 µg l^−1^; Table 2) and condensate (107 µg l^−1^)^29^ WAFs. The sensitivity of coral larvae observed here is difficult to compare with other studies, which reported different exposure types, endpoints and measures of petroleum hydrocarbon concentration in WAFs (summarised in Negri, *et al*.^29^ and Turner, *et al*.^2^). Nevertheless, studies exposing coral larvae to hydrocarbons in the absence of UVR reported negative effects on coral settlement at THC concentrations between 82 and 620 µg l^−1^ ^7,33,40^ and the sensitivity of coral larvae is likely to be greater than that of other life stages^2^.

The toxicity of both HFO and diesel WAFs doubled (EC_50_: 95 to 51 and 1300 to 494 µg l^−1^ TPAH ±UVR, respectively) in the presence of ecologically relevant intensities of UVR. This observed phototoxicity is consistent with the 2.5-fold increase in toxicity of natural gas condensate WAF in the presence of UVR to the same larval species in 24 h WAF exposures, based on measured TPAH concentrations^29^. This toxicity increase is similar between studies despite differences in UVR exposure profiles, where Negri, *et al*.^29^ applied a single, higher intensity UVR dose for a shorter period of time (2 h, summing to 39 W cm^−2^) compared to this study (see Methods). Overmans *et al*.^18^ recently reported that the inhibition of coral larval metamorphosis by the PAH anthracene increased 7-fold in the presence of UVR (EC_50_: 45 −UVR to 6.3 +UVR µg l^−1^), while there was little influence of UVR exposure on the toxicity of phenanthrene. The impacts of PAH and UVR on larval metamorphosis were also detected at lower PAH concentrations than other sub-lethal biomarkers investigated following the 48 h exposures (including 10 h UVR exposure per day)^18^. Peachey and Crosby^17^ observed 100% mortality of *F*. *scutaria* larvae after 1 h exposures to 48 µg l^−1^ pyrene followed by exposure to ambient solar radiation (including UVR). Strong phototoxic effects have also been demonstrated for adult *P*. *varicata* corals where UVR increased the toxicity of fluoranthene by approximately 14-fold within 72 h following a 4.5 h exposure to this PAH^27^. It is not clear whether the main influence of UVR is to generate more toxic transformation products, or whether the additional toxicity is caused by elevated oxidative stress within the larvae as PAHs, excited by UVR, decay back to their ground states (photosensitisation)^12,13^. However, phototoxicity in aquatic organisms is generally considered to be caused by photosensitisation^18^. While UVR increase the toxicity of some individual PAHs by more than 10 times, fuel WAFs are comprised of many aromatics and several of these, including BTEX, the naphthalenes and phenanthrene, are not considered phototoxic^41^. Consequently, very large increases in oil or fuel WAF toxicity in the presence of UVR are unlikely.

In addition to reductions in settlement success, exposure to fuel WAFs also caused changes to larval morphology and development. Slowed onset and completion of metamorphosis, as well as increased instances of morphological abnormalities, were observed in larvae exposed to WAFs. The process of attachment and metamorphosis following addition of settlement inducers was slower at moderate-high TPAH concentrations, with only partial metamorphosis achieved in 24 h, similar to what would generally be expected 6–12 h after settlement induction (A.J. Heyward, personal communication, Dec 2016). Delayed onset of metamorphosis, abnormal development or deformations following exposures to petroleum hydrocarbons have also been reported for larvae and juvenile polyps from other coral species^28,29,42^. The development of abnormal morphologies in coral larvae may indicate narcosis or more specific toxic effects of petroleum hydrocarbons on cellular developmental processes.

The results of this study clearly demonstrate the strong phototoxic effects of the water-soluble fractions of common marine fuels on coral larval settlement in the presence of environmentally realistic UVR exposures. The current study applied UVR intensities slightly lower than the intensities measured at 1 m depth on a mid-shelf (Trunk Reef) and inshore reef (Esk reef) on the Central GBR. These intensities are also consistent with previous measurements on the GBR^23^, and other coastal and oceanic locations^22,24^. The penetration of UVR in marine environments is primarily affected by chromophoric dissolved organic matter (CDOM) and, for some systems, particulate matter such as plankton and detritus^22,24^. Attenuation is therefore likely to be lowest in clear-water oligotrophic coral reef environments, as measured at the mid-shelf site at Trunk Reef, compared to the more turbid waters of the inshore Esk Reef. Barron *et al*.^20^ found that at depths greater than 0.5 m attenuation of light varies between habitats, emphasising the need to accurately estimate the intensity and wavelengths experienced by each species or ecosystem when investigating the influence of UVR on the toxicity of pollutants. Coral gametes, embryos and larvae developing at the water surface may also be exposed to substantially higher UVR intensities *in situ* than applied in the present study, potentially reducing the toxic threshold values further. Moreover, these results suggest that the hazard (hence risk) posed by aqueous petroleum hydrocarbons to shallow-water tropical coral reefs will be underestimated if phototoxic activation by UVR is not taken into consideration.

The NTLM for predicting narcotic mortality underestimated the impacts of HFO WAF in comparison to the observed effects on larval settlement. 50% larval mortality was predicted for HFO WAFs by the NTLM at ~900 µg l^−1^ TPAH (TU_Neat fuel_); however, coral larval settlement was reduced by 50% at far lower concentrations (EC_50_s 96 to 51 µg l^−1^ TPAH ±UVR). It has been reported that the NTLM can underestimate the toxicity of some hydrocarbon mixtures^14^, and these underestimations may result from the assumption of a simple narcotic mode of action for all aromatic components. The observed discrepancies could partially result from the contribution of other toxic mechanisms, especially those that specifically affect coral metamorphosis from pelagic planula to sessile juveniles, as suggested by Negri *et al*.^29^. Furthermore, the sub-lethal endpoint of settlement inhibition (while ecologically relevant^36^) is by definition more sensitive than mortality, which is typically used to generate the CTLBB values used in the NTLM. Determining CTLBB values for both larvae and adults of key coral reef species would improve species-specific toxicity predictions at different life stages and assist in ranking the potential risks posed by crude oils and petroleum products^15^. It is also possible that other components (e.g. photoproducts) that were not measured may have influenced the toxicity. Although a phototoxic target lipid model was recently presented^30^, its use is currently limited to estimation of the acute toxicity of individual PAHs, and further development is necessary before it can be applied to complex hydrocarbon mixtures or chronic exposures.

Even in well managed and protected areas, groundings of large vessels and petroleum hydrocarbon releases from offshore extraction facilities have occurred in the last decade^43–45^. Large spills as well as chronic hydrocarbon pollution can lead to degradation of adult coral health and changes to reef composition^7,46,47^ with recovery taking more than 10–20 years^1^. The slow recovery of these reefs is likely to be at least partially due to long term effects of hydrocarbon contamination on recruitment processes, including larval settlement^48^; used as a sensitive toxicity endpoint in this study. The overlap between a large oil spill and the coral recruitment window can be substantial, with water from some spill sites remaining phototoxic to invertebrate embryos for up to 13 d^49^ and larvae of some coral species remaining in the water column for up to 3 months (as reviewed by Jones, *et al*.^50^). The increase in toxicity of dissolved aromatics from HFO by UVR exposure resulted in low toxic thresholds, underscoring the potential hazard to corals posed by phototoxic compounds found in petroleum oils and fuels. Previous assessments may therefore have substantially underestimated the risks posed by oil and petroleum product spills on shallow-water, tropical coral reefs by not accounting for interactions with environmental factors such as UVR. Further research into the effects of petroleum hydrocarbons on more tropical reef organisms, including potential interactions with UVR and other stressors, is needed to more effectively quantify these risks.

## Methods

### Coral collection and larval cultures

Gravid colonies of *Acropora tenuis*, a reef-building coral common throughout the Pacific Ocean, were collected by hand on SCUBA from Magnetic Island (October 2016, 19.157°S, 146.861°E), GBR, under Great Barrier Reef Marine Park Authority Permit G12/35236.1. Colonies were placed in flow-through seawater of ambient temperature and transported to the National Sea Simulator, Townsville, within 24 hours. On arrival colonies were transferred to 70% shaded flow-through outdoor holding tanks and kept at temperatures equivalent to the collection site (27 °C) until spawning. When showing signs of setting colonies were isolated and gametes collected by gentle scooping.

Larval cultures (6 parental colonies, 95% fertilisation) were initiated on the 19^th^ October 2016. Cultures were maintained at 27 °C and densities <500 larvae per l at ~27 °C^33^ in round, 500 l fibreglass rearing tanks with cone-shaped bases. Flow-through seawater (1.5 turnovers per day) was 1 µm-filtered and a round air stone at the base of each tank provided aeration and created a gentle curtain of bubbles to keep larvae from a submerged cylindrical mesh filter (15 h × 6 d cm, 100 µm) at the outflow.

### Preparation of fuel water accommodated fractions

Heavy residual fuel oil (HFO, International Bunker Supplies Pty Ltd, Gladstone, Australia) and automotive diesel (Puma Energy Australia, Fortitude Valley, Australia) WAFs were prepared in capped, solvent rinsed aspirator bottles (5 l) using 0.45 µm filtered seawater (FSW; pH 8.1, salinity 37.0 psu) loaded at 20 g fuel l^−1^ with a 20% headspace^51^. Solutions were protected from light, stirred at 50 rpm for 18 h without a vortex and allowed to settle for 30 min prior to use^51^. Ten dilutions (0, 0.4, 0.8, 1.6, 3.125, 6.25, 12.5, 25, 50 and 100% WAF) were prepared from the fresh undiluted (100%) WAF using 0.45 µm FSW^29,52^ and used within 3 h in the assays below.

### Fuel phototoxicity assays

*Acropora* spp. larvae reach competency to settle and undergo metamorphosis after ~4 d^50^. HFO and diesel static non-renewal exposure experiments over 48 h were performed on 7- and 8-d old *A*. *tenuis* larvae, respectively. 10–14 larvae and 20 ml of WAF were gently added to 6 replicate glass vials for each WAF concentration and ±UVR treatment combination (12 replicate vials for 0% WAF controls). Vials were tightly capped with approximately 10% headspace^51^ to allow for oxygen exchange. Vials were randomly placed on their side in trays inside temperature controlled, orbital shaker incubators (Thermoline Scientific, Australia) at 80 rpm to ensure larvae did not settle during the exposure period. One set of vials (−UVR), n = 66, was placed in an incubator fitted with actinic LEDs emitting 1.23 mW cm^−2^ photosynthetically active radiation (PAR; Aqualina Blue 450 nm, 10,000 K and 420 nm Actinic LED strips, The Aquatic Life Product Company, Willawong, Australia). The second set of vials (n = 66) was placed in a second incubator in the presence of both actinic light and UVR (+UVR). This incubator (+UVR) was fitted with identical actinic LEDs as the first incubator (PAR: 0.95 mW cm^−2^) as well as three sets of UV-emitting fluorescent tubes (each set consisting of one Deluxlite Blacklight Blue 18 W and one Reptile One UVB 5.0 18 W T8 fluorescent tube) emitting 0.75 mW cm^−2^ UVR. Fluorescent tubes emitting predominantly in the UVA spectrum were chosen due to the high UVB attenuation of seawater^19^ (See Ultraviolet radiation intensities section below on how fluorescent tube irradiance was characterised, and Fig. 1c for comparison of experimental lights, light attenuation of vials used for experimental exposures and *in situ* intensities of UVR on the GBR). PAR was provided on a 12:12 h L:D cycle and UVR on a 6:18 h L:D cycle (total irradiance 16.1 W cm^−2^ d^−1^). An additional 30 vials containing undiluted WAF, but no larvae, were also placed in each incubator to allow for the collection of chemical samples at the end of the 48 h exposure. The positions of vials within each incubator were exchanged randomly throughout each experiment to minimize variation in light exposure. Temperature was continuously logged (Onset HOBO temperature logger, Onset Computer Corp., Massachusetts, USA) while pH, salinity and dissolved oxygen concentrations were measured at the beginning and end of each experiment. At the end of the 48 h exposure approximately 600 ml of undiluted WAF was pooled, from the 30 additional vials (not containing larvae), for chemical analysis (see Chemical analysis below).

Following exposure to fuel WAFs in the presence or absence of UVR, larvae were transferred with 10 ml treatment solution directly to individual wells in 6-well cell culture plates (Nalge Nunc Int., Denmark). Larvae were presented with a settlement inducer consisting of 5 × 5 mm live chips of the crustose coralline algae (CCA) *Porolithon onkodes*^53^. Settlement success was assessed as percentage of larvae which attached and underwent at least early metamorphosis (i.e. became firmly attached and flattened into a disc shape^53^) after ~24 h incubation at 27 °C. Average settlement success ≥70% in controls was considered indicative of a normal response to settlement inducers based on several previous studies using CCA or extracts of CCA to initiate settlement^29,33,37,54,55^. Additional notes regarding deformities, ratio of fully (as outlined by Heyward & Negri^53^) to partially metamorphosed recruits as well as failure to attach were also made for each treatment combination.

### Settlement data analysis

Settlement data was fitted with binomial generalized linear models (GLMs) with a logit link function using the R stats-package (R version 3.4.1^56^) to model the settlement success of *A*. *tenuis* larvae in response to treatment concentration (fixed numerical factor) for each light treatment and fuel type combination. The fitted models were validated by plotting the simulated residuals against fitted values^57^ and ensuring no individual values influenced the model fits’ disproportionally. Quasibinomial GLMs were fitted where diagnostics indicated overdispersion. An r^2^ analogue was calculated using the deviance of the fitted models with and without the fixed numerical factor (equation (1)) to approximate the goodness-of-fit for each model^58^.

1 $$Approximate\,\,{r}^{2}=1-(\frac{devianc{e}_{Full{model}}}{devianc{e}_{Null{model}}})$$

EC_10_ and EC_50_ values with 95% confidence intervals (CI) were interpolated from model mean values and 95% CI (adapted from Venables & Ripley^59^). Predicted model mean values and 95% CI were exported and graphical outputs produced using GraphPad Prism (version 7.02, GraphPad Software Inc., CA, USA). The high levels of replication used allowed the identification of outliers in the dataset which were excluded. These comprised three HFO FSW controls and one diesel low concentration replicate where CCA chips induced 0% and 7% settlement, respectively, likely due to misidentification of a few of the live CCA chips.

### Chemical analysis

Samples of freshly prepared, undiluted WAF (“100% WAF”) were collected for chemical analysis at the beginning of each experiment (t_0h_). Undiluted WAF was also added to 30 vials per UVR treatment and incubated simultaneously with vials containing larvae to ensure that the undiluted WAF was exposed to the same experimental conditions as the test solutions. At the end of the exposure period (t_48h_), the undiluted WAF, in vials containing no larvae, was pooled and sampled for chemical analysis. For BTEX analysis, samples (40 ml) were collected in amber glass vials with open hole caps and PTFE septa. For all other analyses, single samples (500 ml) were collected in amber glass bottles with PTFE-lined caps. All samples were acidified to pH 2 using 6 M hydrochloric acid and stored at 4 °C until shipped to ChemCentre (Perth, Australia) for analysis as previously described by Negri *et al*.^29^. Briefly, WAFs were analysed directly for BTEX using Purge and Trap GC-MS in full scan mode (USEPA method 8260). The 500 ml WAF samples were extracted three times with dichloromethane (DCM) and the combined extracts analysed for PAH and alkylated PAH using GC-MS in SIM or scan mode, and TRH using GC-FID (USEPA method 8270). Neat HFO and diesel were diluted in DCM and analysed for TRH, BTEX, PAH/alkylated PAH and phenols using the same methodology, except additional surrogate (2-fluorophenol, phenol-d5 and 2,4,6-tribromophenol) and internal (1,4-dichlorobenzene-d4) standards were added to the samples prior to analysis. To profile their general composition, the neat oils (1 µl) were subjected to whole oil analysis using GC-MS and hydrocarbons were identified through comparison with a pre-characterised reference oil.

### Ultraviolet radiation intensities

*In situ* UVR irradiance on the Central GBR during spring was assessed through full spectrum measurements of UVR in air and at five depths at two reefs. Esk Reef (18.775°S 146.522°E), located in the Palm Islands-group (QLD), was selected as a representative inshore reef site while Trunk Reef (18.329°S, 146.846°E) was selected as a representative clear-water, mid-shelf reef site. Three replicate light intensity measurements for wavelengths between 300–400 nm were performed on SCUBA using a Jaz handheld spectrometer (Ocean Optics, Inc., Florida, USA) and a 5 m fibre optic cable (CPATCH-5074768, Ocean Optics, Inc., Florida, USA) with a planar irradiance collector for underwater use (HOBI Labs, Inc., USA) following Shick *et al*. (1996). Measurements were made with the sensor positioned vertically at 0, 0.1, 1, 2, 3 and 3.8 m depth. Measurements were performed close to solar noon on the mid-shelf (14:17–14:20 on 12 October 2016) and inshore reefs (13:55–14:04 on 14 October 2016), respectively. Intensity data collected was used to calculate the average relative decrease in radiation for wavelengths between 300–400 nm with depth. Total UVA and UVB radiation above the surface was recorded (Solarmeter Model 5.0 UVA + UVB meter, Solartech Inc., Pennsylvania, USA) and turbidity measurements were performed (90FL-T, TPS Pty Ltd, Brendale, QLD, Australia). Cloud coverage was low (<5%) with 17 km/h E winds (BOM, 2016) and turbidity of 0.1 NTU during mid-shelf measurements with medium-high cloud coverage (~80%), 33 km/h ENE winds (maximum gusts 46 km/h ENE; BOM, 2016b) and 0.8 NTU turbidity during inshore reef measurements. Theoretical irradiance at each depth was calculated using full spectrum measurements of natural sunlight close to solar noon on a low cloud coverage day (<5%). Measurements were made using a calibrated Jaz spectrometer and a 250 mm UVR compatible fibre optic cable (QP600-025-UV, Ocean Optics, Inc., Florida, USA) aimed directly towards the sun. Z10% values at 305 and 340 nm were estimated by calculating the irradiance corresponding to 10% of surface irradiance (in air) for each reef site and the negative linear relationship between irradiance and measurement depth of measurements made at Trunk Reef and Esk Reef.

Full spectrum measurements of radiation emitted by the UVR fluorescent tubes (Deluxlite Black Light Blue 18W; Reptile One UVB 5.0 18W), used in +UVR larval settlement assays, were performed using the same calibrated Jaz spectrometer and 250 mm UVR compatible fibre optic cable used to quantify the UV radiation of natural sunlight. Full spectrum measurements were made at approximately the same distance as sample vials during experimental exposures (170 mm) in five separate positions relative to the three sets of fluorescent tubes. Total UVA and B radiation measurements were also performed (Solarmeter Model 5.0 UVA + UVB meter, Solartech Inc., Pennsylvania, USA) for the five replicates. Additionally, the attenuation of UVR, between 300–400 nm, emitted from fluorescent tubes by scintillation vial glass was estimated (calibrated Jaz spectrometer). Measurements were made 200 mm from UVR fluorescent tubes (Deluxlite Black Light Blue 18W; Reptile One UVB 5.0 18W) through the base of a 20 ml scintillation vial. The average total UVA and UVB exposure of larvae inside scintillation vials was calculated using measurements performed with the Solarmeter model 5.0 and the average attenuation of scintillation vial glass between 300–400 nm.

### Toxic unit calculations for narcotic toxicity

The toxicity of aromatics to aquatic organisms is dependent on the partitioning of dissolved compounds between water and lipids. The narcotic toxicity of hydrocarbon mixtures can be estimated using the narcotic target lipid model (NTLM) which combines the octanol-water partitioning coefficients (K_OW_) of all dissolved components in the solution, their measured (or expected) aqueous concentrations and the critical lipid body burden (CTLBB) of the organism of interest (where the CTLBB is the amount of the compound dissolved in the organism’s tissues which causes a specific toxic effect; e.g. 50% mortality)^14^. The NTLM is useful for assessing the relative risks posed by different oil and fuel types to aquatic organisms, but may be less useful if the CTLBB is unknown or may underestimate toxicity if other toxic modes of action are important for a given species^60^.

The dissolved composition of petroleum hydrocarbons in a WAF can be measured (Table S-2, Supplementary information) or modelled by applying an oil solubility model (e.g. PETROTOX) to an oil of known composition^39^. Toxic units (TU) for each constituent in a WAF are defined as the ratio between the concentration (C_WAF, i_) and the 50% critical effect level (LC_50,*i*_, EC_50,*i*_ or IC_50,*i*_) of each constituent (*i*) in a solution, and can be used to enable comparisons of studies using differing experimental designs and hydrocarbon compositions^15^. Assuming the same mode of action (narcosis) for all MAHs and PAHs, the TUs for each component of a WAF containing a mixture of PAHs and MAHs, are considered additive and can be summed to estimate the TU of the WAF^32^ according to equation (2).

2 $$T{U}_{WAF}=\sum \frac{{C}_{WAF,1}}{E{C}_{50,1}}+\frac{{C}_{WAF,2}}{E{C}_{50,2}}+\frac{{C}_{WAF,3}}{E{C}_{50,3}}+\cdots +\frac{{C}_{WAF,i}}{E{C}_{50,i}}$$

A TU of 1 indicates that the mixture is predicted to be toxic and will affect 50% of exposed organisms^32^. In this study, expected TUs (TU_Neat fuel_) were calculated as per Redman and Parkerton^15^, by applying the observed concentrations of individual compounds in neat HFO and diesel and the average LC_50_ CTLBB (86.8 µmol g^−1^ octanol) of 15 marine and estuarine organisms^14,61^. An average marine CTLBB was used as no CTLBB currently exist for acroporid corals. Additionally, observed TUs (TU_WAF_) for each light treatment were calculated using measured concentrations of individual compounds in undiluted HFO and diesel WAFs and the same CTLBB value.

### Data availability

Data are available upon request.

## Electronic supplementary material

Supplementary information
