# Supplementary material for: Phototoxic effects of two common marine fuels on the settlement success of the coral Acropora tenuis
Source: Sci Rep. 2018 Jun 5;8:8635. doi: 10.1038/s41598-018-26972-7 (PMC5988723; doi:10.1038/s41598-018-26972-7)
Supplement: Supplementary file 1 — Supplementary information [file 41598_2018_26972_MOESM1_ESM.pdf]

Phototoxic effects of two common marine fuels on the settlement success of the coral *Acropora tenuis*

F. Mikaela Nordborg[1, 2, *], Florita Flores[1], Diane L. Brinkman[1], Susana Agusti[3], Andrew P. Negri[1, 4]

[1]Australian Institute of Marine Science, Townsville, 4810, Queensland, Australia

[2]Department of Marine Sciences, University of Gothenburg, Göteborg, 40530, Sweden

[3] Red Sea Research Centre, King Abdullah University of Science and Technology, Biological Environmental Science and Engineering Division, Thuwal, 23955, Saudi Arabia

[4] AIMS@JCU, Division of Research & Innovation, James Cook University and Australian Institute of Marine Science, Townsville, 4810, Queensland, Australia

*F.M.N. email: m.nordborg@aims.gov.au

Table S-1. Chemical composition of neat heavy fuel oil and diesel. Analysis performed by ChemCentre (Perth, Australia). LOR = limit of reporting, BTEX = benzene, toluene, ethylbenzene and xylenes, PAH = polycyclic aromatic hydrocarbon, TPAH = total petroleum aromatic hydrocarbon, TRH = total recoverable hydrocarbons.

Analyte	Method Code	LOR	Heavy Fuel Oil (mg kg^{-1})	Automotive Diesel (mg kg^{-1})
Benzene	ORG002S	0.2	6.9	41
Toluene	ORG002S	0.2	40	350
Ethylbenzene	ORG002S	0.4	25	200
m,p-Xylene	ORG002S	0.2	83	580
o-Xylene	ORG002S	0.2	35	320
Naphthalene	ORG020P	10	1500	240
C1-alkylnaphthalenes	ORG020P	20	6600	420
C2-alkylnaphthalenes	ORG020P	100	9800	1100
C3-alkylnaphthalenes	ORG020P	100	7800	1800
C4-alkylnaphthalenes	ORG020P	100	2500	960
Acenaphthylene	ORG020P	10	<20	<20
Acenaphthene	ORG020P	10	160	<20
Fluorene	ORG020P	10	200	<20
Dibenzothiophene	ORG020P	20	170	<20
C1-alkyldibenzothiophenes	ORG020P	100	940	<100
C2-alkyldibenzothiophenes	ORG020P	100	1700	<100
C3-alkyldibenzothiophenes	ORG020P	100	1500	<100
Phenanthrene	ORG020P	10	480	43
Anthracene	ORG020P	10	83	<20
C1-alkylphenanthrenes	ORG020P	100	2200	330
C2-alkylphenanthrenes	ORG020P	100	2800	420
C3-alkylphenanthrenes	ORG020P	100	3200	410
Fluoranthene	ORG020P	10	<20	<20
Pyrene	ORG020P	10	160	<20
C1-alkylpyrenes/fluoranthenes	ORG020P	100	1100	<100
C2-alkylpyrenes/fluoranthenes	ORG020P	100	1400	<100
C3-alkylpyrenes/fluoranthenes	ORG020P	100	1500	<100
Benz(a)anthracene	ORG020P	10	90	<20
Chrysene	ORG020P	10	310	<20
C1-alkylchrysenes	ORG020P	100	980	<100
C2-alkylchrysenes	ORG020P	100	1000	<100
Benzo(b)fluoranthene	ORG020P	10	<20	<20
Benzo(k)fluoranthene	ORG020P	10	<20	<20
Benzo(a)pyrene	ORG020P	10	81	<20
C1-alkylbenzopyrenes	ORG020P	100	690	<100
C2-alkylbenzopyrenes	ORG020P	100	1500	<100
Indeno(1,2,3-cd)pyrene	ORG020P	10	<20	<20
Dibenzo(a,h)anthracene	ORG020P	10	<20	<20
Benzo(g,h,i)perylene	ORG020P	10	50	<20
2-methylphenol	ORG100S	10	<200	<200
2,3,4,6-Tetrachlorophenol	ORG100S	10	<200	<200
2,4,5-Trichlorophenol	ORG100S	10	<200	<200
2,4,6-Trichlorophenol	ORG100S	10	<200	<200
2,4-Dichlorophenol	ORG100S	10	<200	<200
2,4-Dimethylphenol	ORG100S	10	<200	<200
2,6-Dichlorophenol	ORG100S	10	<200	<200
2-Chlorophenol	ORG100S	10	<200	<200
2-Nitrophenol	ORG100S	10	<200	<200
3- & 4-Methylphenol	ORG100S	10	<200	<200

4-Chloro-3-methylphenol	ORG100S	10	<200	<200
Pentachlorophenol	ORG100S	10	<200	<200
Phenol	ORG100S	10	<200	<200
**∑BTEX**			**190**	**1491**
**∑PAH**			**50494**	**5723**
**TPAH**			**50684**	**7214**
TRH C6-C10	ORG007P	25	16000	50000
TRH >C10-C16	ORG007P	25	97000	450000
TRH >C16-C34	ORG007P	100	200000	370000
TRH >C34-C40	ORG007P	100	10000	670
**∑TRH**			**320000**	**870000**

Figure S-1. Chromatogram showing composition of heavy fuel oil (HFO).

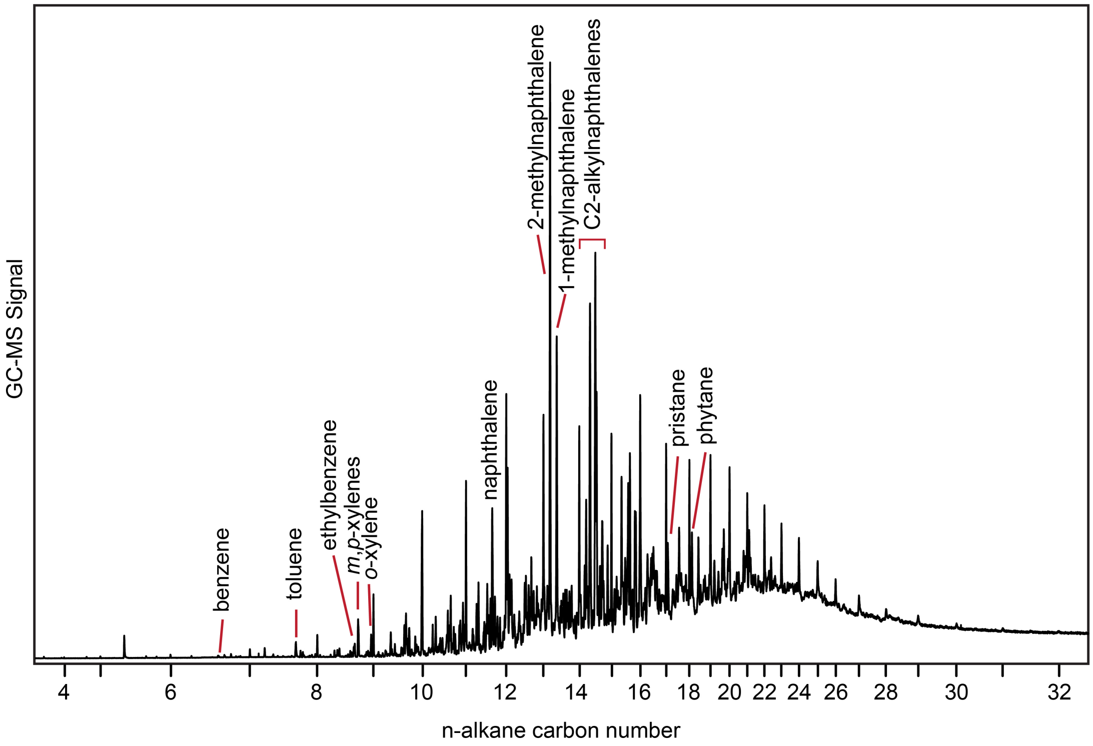

Figure S-2. Chromatogram showing composition of automotive diesel.

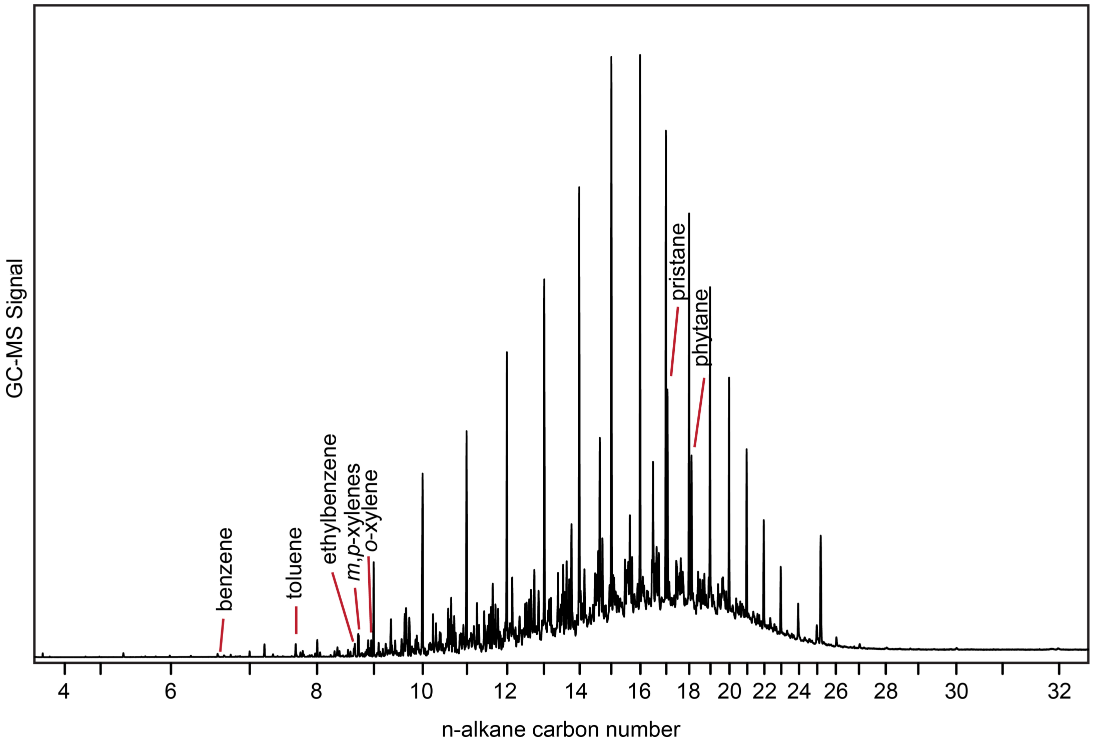

Table S-2. Chemical composition of heavy fuel oil and diesel WAFs. Analysis performed by ChemCentre (Perth, Australia). LOR = limit of reporting, BTEX = benzene, toluene, ethylbenzene and xylenes, PAH = polycyclic aromatic hydrocarbon, TPAH = total petroleum aromatic hydrocarbon, TRH = total recoverable hydrocarbons.

			Heavy Fuel Oil (µg l^{-1})			Automotive Diesel (µg l^{-1})		
**Analyte**	**Method Code**	**LOR**	$t_{0\,h}$	$t_{48\,h}$ -UVR	$t_{48\,h}$ +UVR	$t_{0\,h}$	$t_{48\,h}$ -UVR	$t_{48\,h}$ +UVR
Benzene	ORG015W	1	71	71	74	130	88	91
Toluene	ORG015W	1	170	170	170	340	240	250
Ethylbenzene	ORG015W	1	43	37	37	73	44	49
m,p-Xylene	ORG015W	2	130	150	120	210	130	150
o-Xylene	ORG015W	1	74	79	66	140	94	100
Naphthalene	ORG020W	0.1	170	160	140	8.7	4.8	5.5
C1-alkylnaphthalenes	ORG020W	0.1	184	195	171	5.3	2.0	2.6
C2-alkylnaphthalenes	ORG020W	0.5	75	72	66	3.5	1.6	2.0
C3-alkylnaphthalenes	ORG020W	0.5	7.8	6.4	5.5	1.7	<1.0	1.2
C4-alkylnaphthalenes	ORG020W	0.5	1.4	1.3	1.0	<1.0	<1.0	<1.0
Acenaphthylene	ORG020W	0.1	<0.2	<0.2	<0.2	<0.2	<0.2	<0.2
Acenaphthene	ORG020W	0.1	0.9	0.8	0.7	<0.2	<0.2	<0.2
Fluorene	ORG020W	0.1	1.1	0.8	0.7	0.3	<0.2	0.3
Dibenzothiophene	ORG020W	0.1	0.4	0.4	0.3	<0.2	<0.2	<0.2
C1-alkyldibenzothiophenes	ORG020W	0.5	<1.0	<1.0	<1.0	<1.0	<1.0	<1.0
C2-alkyldibenzothiophenes	ORG020W	0.5	<1.0	<1.0	<1.0	<1.0	<1.0	<1.0
C3-alkyldibenzothiophenes	ORG020W	0.5	<1.0	<1.0	<1.0	<1.0	<1.0	<1.0
Phenanthrene	ORG020W	0.1	0.9	0.9	0.7	<0.2	<0.2	<0.2
Anthracene	ORG020W	0.1	<0.2	<0.2	<0.2	<0.2	<0.2	<0.2
C1-alkylphenanthrenes	ORG020W	0.5	<1.0	<1.0	<1.0	<1.0	<1.0	<1.0
C2-alkylphenanthrenes	ORG020W	0.5	<1.0	<1.0	<1.0	<1.0	<1.0	<1.0
C3-alkylphenanthrenes	ORG020W	0.5	<1.0	<1.0	<1.0	<1.0	<1.0	<1.0
Fluoranthene	ORG020W	0.1	<0.2	<0.2	<0.2	<0.2	<0.2	<0.2
Pyrene	ORG020W	0.1	<0.2	<0.2	<0.2	<0.2	<0.2	<0.2
C1-alkylpyrenes/fluoranthenes	ORG020W	0.5	<1.0	<1.0	<1.0	<1.0	<1.0	<1.0
C2-alkylpyrenes/fluoranthenes	ORG020W	0.5	<1.0	<1.0	<1.0	<1.0	<1.0	<1.0
C3-alkylpyrenes/fluoranthenes	ORG020W	0.5	<1.0	<1.0	<1.0	<1.0	<1.0	<1.0
Benz(a)anthracene	ORG020W	0.1	<0.2	<0.2	<0.2	<0.2	<0.2	<0.2
Chrysene	ORG020W	0.1	<0.2	<0.2	<0.2	<0.2	<0.2	<0.2
C1-alkylchrysenes	ORG020W	0.5	<1.0	<1.0	<1.0	<1.0	<1.0	<1.0
C2-alkylchrysenes	ORG020W	0.5	<1.0	<1.0	<1.0	<1.0	<1.0	<1.0
Benzo(b)fluoranthene	ORG020W	0.1	<0.2	<0.2	<0.2	<0.2	<0.2	<0.2
Benzo(k)fluoranthene	ORG020W	0.1	<0.2	<0.2	<0.2	<0.2	<0.2	<0.2
Benzo(a)pyrene	ORG020W	0.1	<0.2	<0.2	<0.2	<0.2	<0.2	<0.2
C1-alkylbenzopyrenes	ORG020W	0.5	<1.0	<1.0	<1.0	<1.0	<1.0	<1.0
C2-alkylbenzopyrenes	ORG020W	0.5	<1.0	<1.0	<1.0	<1.0	<1.0	<1.0
Indeno(1,2,3-cd)pyrene	ORG020W	0.1	<0.2	<0.2	<0.2	<0.2	<0.2	<0.2
Dibenzo(a,h)anthracene	ORG020W	0.1	<0.2	<0.2	<0.2	<0.2	<0.2	<0.2
Benzo(g,h,i)perylene	ORG020W	0.1	<0.2	<0.2	<0.2	<0.2	<0.2	<0.2
∑BTEX			**488**	**507**	**467**	**893**	**596**	**640**
∑PAH			**442**	**438**	**386**	**20**	**8**	**12**
TPAH			**930**	**945**	**853**	**913**	**604**	**652**
TRH C6-C10	ORG007W	25	760	510	590	1400	990	830
TRH >C10-C16	ORG007W	50	1200	1100	1100	520	500	360
TRH >C16-C34	ORG007W	100	<100	<100	<100	<100	<100	<100
TRH >C34-C40	ORG007W	100	<100	<100	<100	<100	<100	<100
∑TRH			**1960**	**1610**	**1690**	**1920**	**1490**	**1190**

Table S-3. Predicted chemical composition of heavy fuel oil and diesel WAFs. Calculated as per Redman & Parkerton (2015) from neat heavy fuel oil and automotive diesel compositions (Table S-1). BTEX = benzene, toluene, ethylbenzene and xylenes, PAH = polycyclic aromatic hydrocarbon, TPAH = total petroleum aromatic hydrocarbon.

Analyte	Heavy Fuel Oil ($\mu g\ l^{-1}$)	Automotive Diesel ($\mu g\ l^{-1}$)
Benzene	29.6	176.1
Toluene	20.0	174.8
Ethylbenzene	33.8	270.6
m,p-Xylene	36.5	255.5
o-Xylene	14.1	129.3
Naphthalene	293.2	47.0
C1-alkylnaphthalenes	126.0	9.7
C2-alkylnaphthalenes	257.0	24.5
C3-alkylnaphthalenes	40.6	9.4
C4-alkylnaphthalenes	3.7	1.4
Acenaphthylene	15.0	0.0
Acenaphthene	0.0	0.0
Fluorene	3.6	0.0
Dibenzothiophene	0.5	0.0
C1-alkyldibenzothiophenes	0.7	0.0
C2-alkyldibenzothiophenes	0.4	0.0
C3-alkyldibenzothiophenes	0.1	0.0
Phenanthrene	2.3	0.2
Anthracene	0.4	0.0
C1-alkylphenanthrene/anthracenes	3.9	0.6
C2-alkylphenanthrene/anthracenes	1.8	0.3
C3-alkylphenanthrene/anthracenes	0.9	0.1
Fluoranthene	0.0	0.0
Pyrene	0.2	0.0
C1-alkylpyrenes/fluoranthenes	0.7	0.0
C2-alkylpyrenes/fluoranthenes	0.4	0.0
C3-alkylpyrenes/fluoranthenes	0.1	0.0
Benz(a)anthracene	0.0	0.0
Chrysene	0.0	0.0
C1-alkylchrysenes	0.1	0.0
C2-alkylchrysenes	0.1	0.0
Benzo(b)fluoranthene	0.0	0.0
Benzo(k)fluoranthene	0.0	0.0
Benzo(a)pyrene	0.0	0.0
C1-alkylbenzopyrenes	0.0	0.0
C2-alkylbenzopyrenes	0.0	0.0
Indeno(1,2,3-cd)pyrene	0.0	0.0
Dibenzo(a,h)anthracene	0.0	0.0
Benzo(g,h,i)perylene	0.0	0.0
∑BTEX	**134**	**1006**
∑PAH	**752**	**93**
TPAH	**886**	**1099**
